# Sex-Specific Causal Relations between Steroid Hormones and Obesity—A Mendelian Randomization Study

**DOI:** 10.3390/metabo11110738

**Published:** 2021-10-28

**Authors:** Janne Pott, Katrin Horn, Robert Zeidler, Holger Kirsten, Peter Ahnert, Jürgen Kratzsch, Markus Loeffler, Berend Isermann, Uta Ceglarek, Markus Scholz

**Affiliations:** 1 Institute for Medical Informatics, Statistics and Epidemiology, Medical Faculty, University of Leipzig, 04107 Leipzig, Germany; katrin.horn@imise.uni-leipzig.de (K.H.); holger.kirsten@imise.uni-leipzig.de (H.K.); peter.ahnert@imise.uni-leipzig.de (P.A.); markus.loeffler@imise.uni-leipzig.de (M.L.); 2 LIFE Research Center for Civilization Diseases, Medical Faculty, University of Leipzig, 04103 Leipzig, Germany; juergen.kratzsch@medizin.uni-leipzig.de (J.K.); berend.isermann@medizin.uni-leipzig.de (B.I.); Uta.Ceglarek@medizin.uni-leipzig.de (U.C.); 3 Institute of Laboratory Medicine, Clinical Chemistry and Molecular Diagnostics, University Hospital Leipzig, 04103 Leipzig, Germany; robert.zeidler@googlemail.com

**Keywords:** steroid hormones, sexual dimorphism, genome-wide association analysis, Mendelian randomization, coronary artery disease, obesity

## Abstract

Steroid hormones act as important regulators of physiological processes including gene expression. They provide possible mechanistic explanations of observed sex-dimorphisms in obesity and coronary artery disease (CAD). Here, we aim to unravel causal relationships between steroid hormones, obesity, and CAD in a sex-specific manner. In genome-wide meta-analyses of four steroid hormone levels and one hormone ratio, we identified 17 genome-wide significant loci of which 11 were novel. Among loci, seven were female-specific, four male-specific, and one was sex-related (stronger effects in females). As one of the loci was the human leukocyte antigen (HLA) region, we analyzed HLA allele counts and found four HLA subtypes linked to 17-OH-progesterone (17-OHP), including HLA-B*14*02. Using Mendelian randomization approaches with four additional hormones as exposure, we detected causal effects of dehydroepiandrosterone sulfate (DHEA-S) and 17-OHP on body mass index (BMI) and waist-to-hip ratio (WHR). The DHEA-S effect was stronger in males. Additionally, we observed the causal effects of testosterone, estradiol, and their ratio on WHR. By mediation analysis, we found a direct sex-unspecific effect of 17-OHP on CAD while the other four hormone effects on CAD were mediated by BMI or WHR. In conclusion, we identified the sex-specific causal networks of steroid hormones, obesity-related traits, and CAD.

## 1. Introduction

Male sex is an independent risk factor for cardiovascular disease (CVD), but the underlying molecular mechanisms are not fully understood. Genome-wide association studies have identified several risk loci in the autosomes [1], but none on chromosome X [2]. This indicates that the observed sex-dimorphism of CVD risk is not primarily driven by gonosomal genetics. Since steroid metabolism is highly sex-specific, a causal relationship to atherosclerosis risk can be hypothesized, but the underlying molecular mechanisms are only partly understood.

There are some studies supporting this relationship: estradiol (E2) was suspected of having a cardio-protective effect before menopause [3,4,5] and levels of dehydroepiandrosterone sulfate (DHEA-S) were found to be different between coronary artery disease (CAD) patients and controls [6] with lower DHEA-S levels associated with higher cardiovascular disease mortality [7]. Despite these correlations, the causality of steroid hormones on CVD has not yet been investigated in detail.

Obesity is a disease defined by excessive fat accumulation that might impair health [8]; it also displays a strong sexual dimorphism in particular with respect to body fat distribution mediated by steroid hormones [9,10]. Two common measures of obesity are body mass index (BMI) and waist-to-hip ratio (WHR). While BMI is highly correlated to the percentage of body fat [11], WHR takes differences in body shape into account. WHR adjusted for BMI (WHRadj) has been shown to be a good predictor of cardiovascular events such as ischaemic heart disease [12]. Recent sex-stratified genome-wide association meta-analyses (GWAMA) of BMI and WHRadj found 346 associated loci of which one third was sex-related, mostly with stronger effects in women [13].

It has been shown that steroid hormone signaling is relevant in adipose tissue (AT) regulation [14], e.g., aldosterone receptor signaling induces abnormal secretion of adipokines [15]. Steroid-hormone-converting enzymes have an effect on AT function [16] and their gene expression in AT changes in response to exercise and diet [17]. A cross-sectional study in adult males showed an association between WHR and sex steroid hormones, including the ratio of testosterone (T) and estradiol (E2) [18]. The T/E2 ratio has been suggested as a parameter of the disturbance of the physiological balance of these hormones and might be more meaningful than the absolute quantities of T and E2 [19].

While the causal link of obesity to CAD is well established [20], the relation between steroid hormones and their effects on BMI, WHR, and CAD is less analyzed regarding causality. A longitudinal analysis found no influence of baseline sex hormone levels on changes in obesity measures, but that body composition might affect hormone levels [18]. Moreover, it has not yet been studied how obesity might mediate the causal effect of hormones on CAD. Here, we attempt to clarify the relationship between steroids, obesity, and CAD by a comprehensive Mendelian randomization (MR) network analysis. Assuming allelic randomization takes place during meiosis and three key assumptions are met, this method estimates the causal effect of life-long small changes of an exposure on an outcome [21].

**Figure 1:**
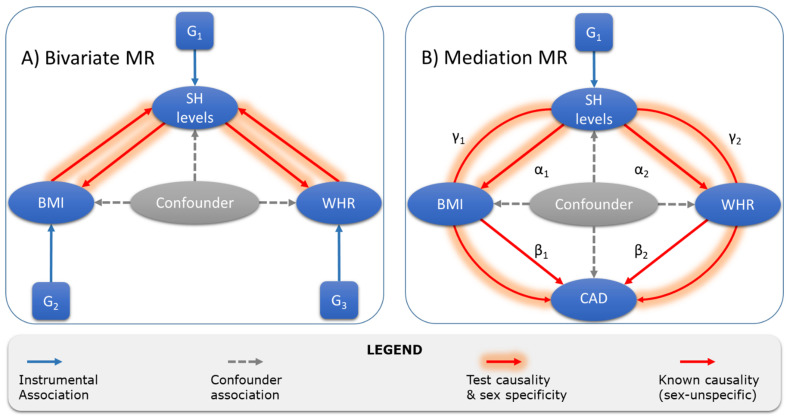
Overview of Mendelian randomization and mediation analyses of the present study. After the identification of valid instruments G_1_ for steroid hormones (SH), we performed two MR analyses. (**A**) First, we conducted bivariate MRs testing causality between SH and the two obesity traits, BMI and WHR. Sex-specific summary statistics and instruments for BMI and WHR were taken from Pulit et al. [13] (G_2_ and G_3_). (**B**) Then, we tested for direct (not shown) and indirect effects (γ_i_) of SH on CAD using causal effect estimates of SH on BMI and WHR (α_i_) and of BMI and WHR on CAD (β_i_, taken from Zhang et al. [20]). CAD summary statistics were obtained from van der Harst et al. [1] (sex-unspecific).

In the present study, we first validate previously published genetic loci associated with steroid hormones [22] and add novel variants as instrumental variables for MR. Then, we use these instruments in bivariate MR to test the causal links between hormones and obesity in both directions. Finally, we examine if there is an effect of steroid hormones on CAD and test whether it is mediated by obesity. A graphical summary of all MR analyses is given in Figure 1.

## 2. Results

### 2.1. GWAMA

To validate known and discover novel instruments for our MR analyses, we performed genome-wide association meta-analyses (GWAMA) of the levels of four hormones, namely progesterone (P4), hydroxyprogesterone (17-OHP), androstenedione (A4), and aldosterone (Aldo) in two independent studies: LIFE-Heart (1357 males, 711 females) [23] and LIFE-Adult (863 males, 618 females) [24]. As the ratio of testosterone to estradiol (T/E2) is suggested a parameter of the disturbance of physiological balance, we analyzed T/E2 as well and searched for additional loci. In Table 1, the baseline characteristics of participants of both studies are given. Genetic data of each study were imputed to 1000 Genomes Phase 3 (European ancestry) [25], and single study association statistics were obtained using the same protocol (see Methods).

**Figure 2:**
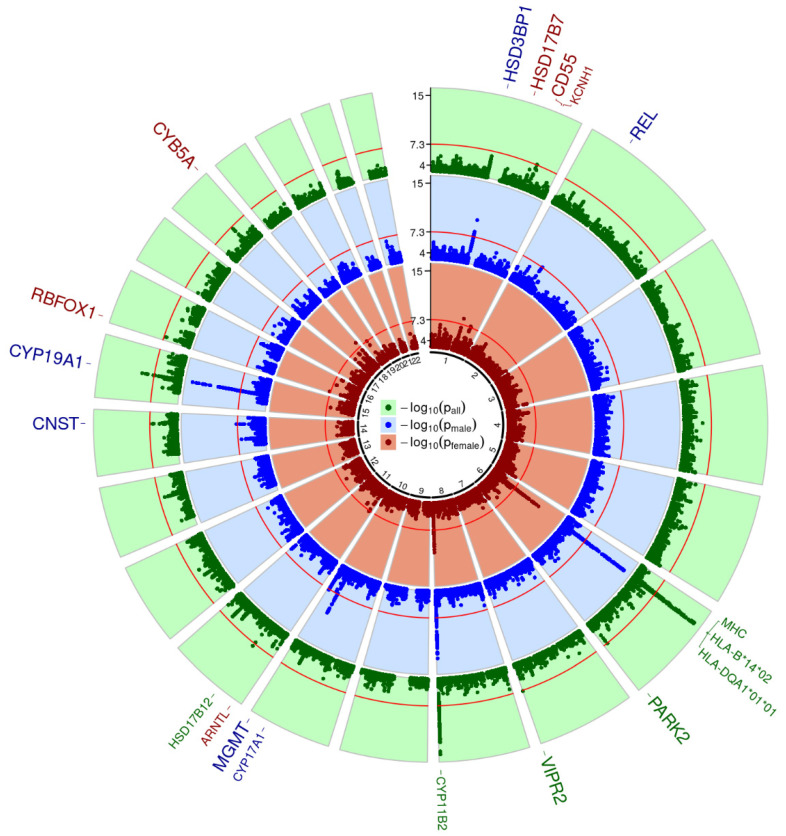
Circular Manhattan plot of GWAMA results. Minimal *p*-values across analyzed traits are presented for each analysis group (green—all samples; blue—male only; red—female only). In each Manhattan circle, the genome-wide significance threshold of 5 × 10^−8^ is given a red circle (−log_10_ transformed). Loci with genome-wide significant SNPs are named outside the circles. They are colored according to the analysis setting showing the highest significance for that locus. Increased font size indicates novelty, while replicated loci are displayed in smaller font size.

We calculated fixed-effect meta-analysis models (FEM) and applied SNP filters for minor allele frequency (MAF, sample size weighted MAF ≥ 1%), imputation info score (minimal score of studies ≥0.8), and heterogeneity of meta-analysis results (I^2^ ≤ 0.9). A total of 10.9 Mio SNPs remained for evaluation, for which we did not observe signs of general inflation of test-statistics (λ in between 0.99 and 1.01). Genome-wide results across all hormones for the combined setting as well as the male- and female-stratified setting are shown in Figure 2. Across all settings and hormones, we detected 35 genome-wide significant and independent SNPs (*p* < 5 × 10^−8^ and pairwise LD r^2^ < 0.1), of which 17 were best-associated in the combined setting, 11 in the male-stratified setting, and 7 in the female-stratified setting. Hits can be summarized to 16 unique genomic loci plus the major histocompatibility complex (MHC) stretching over three cytobands on chromosome 6 (6p21.32, 6p21.33, and 6p22.1). We searched for genetic sex interactions of all genome-wide significant lead SNPs and applied hierarchical FDR correction to adjust for multiple testing (see Methods). Summary statistics, annotations, and interaction results can be found in Tables S1–S5.

**Figure 3:**
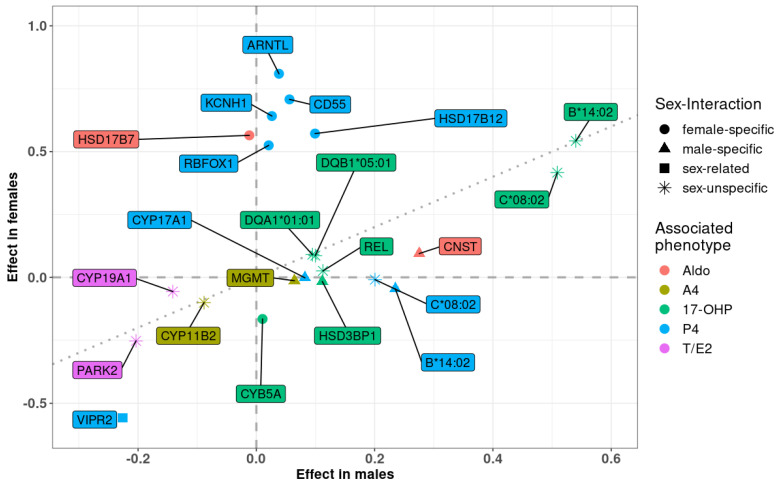
Scatter plot of sex-stratified effect estimates. Types of sex interactions are marked by shape (circle: female-specific; triangle: male-specific; square: sex-related; asterisk: no genetic sex interaction); and loci are colored by associated phenotypes. P4, progesterone; 17-OHP, hydroxyprogesterone; A4, androstenedione; Aldo, aldosterone; T/E2, ratio of testosterone and estradiol.

Eleven of these loci have not yet been described for the respective traits and, hence, are considered as novel findings. For P4, there were three novel loci: *CD55* at 1q32.2 (female-specific) [26], *VIPR2* at 7q36.3 (sex-related, stronger effect in females) [27], and *RBFOX1* at 16p13.3 (female-specific) [28]. For 17-OHP, we also detected three novel hits: *HSD3B1* at 1p12 (male-specific) [22], *REL* at 2p15 (sex-unspecific) [29], and *CYB5A* at 18q22.3 (male-specific) [30]. There was only one novel locus associated with A4: *MGMT* at 10q26.3 (male-specific) [31,32,33]. For the first time, we detected two hits for Aldo: *HSD17B7* at 1q23.3 (female-specific) [22], and in the gene dessert at 14q31.2 (male-specific, trans-eQTL of *CNST*) [34]. Finally, there were two novel loci for T/E2: *CYP19A1* at 15q21.2 (sex-unspecific) [13,22,35], and *PARK2* at 6q26 (sex-unspecific) [36]. A summary of these results is presented in Table 2, and a scatter plot of effect sizes in males and females is shown in Figure 3. A more detailed presentation, including a discussion of plausible candidate genes is provided in the supplemental materials.

### 2.2. Fine-Mapping of the HLA Region

The hormone 17-OHP was associated with multiple variants at the MHC region (872 SNPs with *p* < 1 × 10^−6^), and the other four traits were all associated on a nominal level, e.g., 716 of the 872 SNPs were associated with P4 in males (*p* < 0.05). Therefore, we tested whether the human leukocyte antigen (HLA) subtypes associate with our steroid hormones. HLA subtypes were estimated from imputed genotypes, and associations were calculated using an allele dosage model (see Methods).

**Figure 4:**
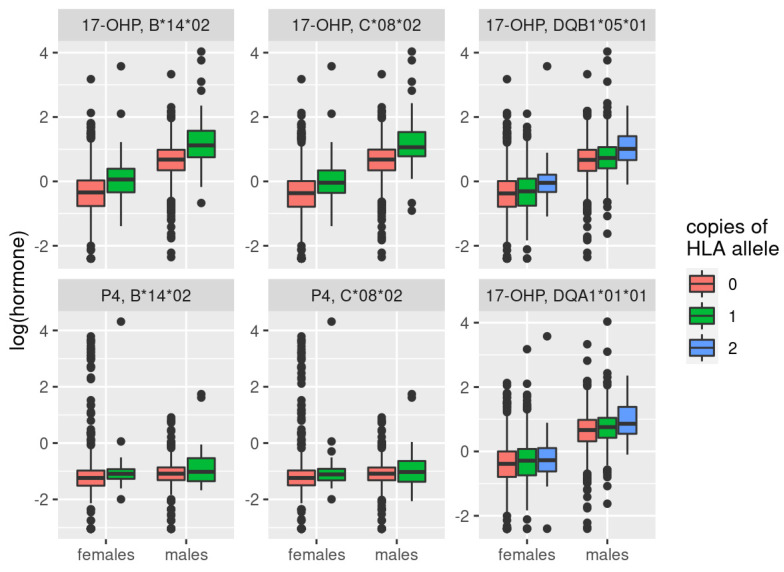
Boxplot of hormone levels for significant associations of HLA subtypes with either 17-OHP or P4. There was only a single male and female sample that had two copies of C*08*02 and B*14*02. For this plot, they were assigned to the group carrying one copy. Data of LIFE-Adult and LIFE-Heart were pooled for this plot, while analyses were performed with a fixed-effect meta-model.

We derived 324 HLA-subtypes in LIFE-Adult and LIFE-Heart. After FDR correction, we detected significant associations for four of them: HLA-C*08*02, HLA-B*14*02, HLA-DQA1*01*01, and HLA-DQB1*05*01 (see Table 3, Table S6 and Figure 4). The first two, HLA-C*08*02 and HLA-B*14*02, are associated with 17-OHP in a sex-unspecific way (q_IA_ = 0.722, q_IA_ = 0.985, respectively). In addition, they were associated with P4 in males, but not in females or the combined setting. The effect difference was only significant for HLA-B*14*02 (q_IA_ = 0.0254), suggesting that this subtype had a male-specific effect on P4, but not on 17-OHP. HLA-C*08*02 and HLA-B*14*02 are in high LD (r^2^ = 0.972 in our data, correlation of estimated subtype allele dosages). We contrasted this observation to measured haplotype frequencies from Wilson et al. [37], where the asymmetric LD (aLD) between HLA-B and -C was estimated as 0.843 (for HLA-C conditioned on HLA-B; 0.650 for HLA-B conditioned on HLA-C). HLA-B*14*02 might be the more plausible candidate here, since it has been linked to *CYP21A1* mutations [38,39].

The other two subtypes (HLA-DQA1*01*01 and HLA-DQB1*05*0) were only associated with 17-OHP in a sex-unspecific way (q_IA_ = 0.985, q_IA_ = 0.985, respectively), and are also in high LD with each other (r^2^ = 0.812 in our data, aLD = 0.819 from [37]). They are only in medium LD with HLA-B and -C (aLD of 0.32 and 0.33, respectively), suggesting a secondary hit next to *CYP21A2*. Both HLA-DQA1 and HLA-DQB1 have been linked to steroid-sensitive nephrotic syndrome [40], and our observed association might provide a missing link between the HLA locus and this syndrome.

### 2.3. Mendelian Randomization

We tested for causal effects of our hormones on obesity-related traits (BMI, WHR) and CAD. Regarding obesity, we also checked for reverse causality and mediation effects on the hormone–CAD link (see Methods). Instruments and summary statistics for BMI, WHR, and CAD were retrieved from the literature [1,13], and the causal estimates for obesity on CAD were taken from [20].

#### 2.3.1. Causal Influence of Steroid Hormones on Obesity-Related Traits

First, we tested for the causal effects of steroid hormones on BMI and WHR, stratified by sex. As instruments, we only used SNPs at loci with biologically plausible genes, e.g., coding for enzymes of the steroid biosynthesis pathway (max dist. 250 kb). There were 13 pairs of hormones and obesity-related traits showing significant causal relationships, of which 12 survived multiple testing corrections (see Table 4, columns “α” and “p(α)” for significant links, and Table S7 for all tested combinations). These comprised five of the nine analyzed hormones (17-OHP, DHEA-S, E2, T, and T/E2), predominantly linked to WHR. For 17-OHP and DHEA-S, instruments for both sexes were available, while the other hormones had only instruments for one of the sexes. For DHEA-S and BMI, we detected sex-related causal effects, with stronger effects in males (p_IA_ = 0.043). The sex-specific effect difference of 17-OHP on WHR did not reach significance (p_IA_ = 0.055).

In an explorative approach, we tested if the results could be replicated using more but weaker SNPs, e.g., considering loci of suggestive significance (*p* < 1 × 10^−6^). We repeated the analyses for all combinations and detected four significant links: E2 on WHR in the combined setting, and, in males, T/E2 on WHR in the combined setting, and 17-OHP on WHR in females. We also repeated the interaction test as, now, instruments were available for both sexes, and found the causal effect of E2 on WHR to be male-specific (p_IA_ = 1.92 × 10^−7^).

We also tested if HLA subtypes could be used as instruments. Here, we considered only 17-OHP and used only HLA-B*14*02 and HLA-DQA1*01*01 so as not to bias the analysis with the correlated instruments. HLA effects on obesity-related traits were estimated in the LIFE studies as summary statistics for HLA associations were not publicly available. We detected a nominally significant causal effect in all three settings on WHR but not BMI, and the interaction test revealed a sex-related effect on WHR, with stronger effects in females (p_IA_ = 7.42 × 10^−3^, see also Table S8).

#### 2.3.2. Test for Reversed Causality of Obesity-Related Traits on Steroid Hormones

To assess whether there is reverse causality of obesity-related traits on steroid hormone levels, we tested these causal directions using genome-wide significant instruments from Pulit et al. [13], allowing only one SNP per cytoband. We observed eight significant causal relationships, including BMI on DHEA-S and WHR on E2 and T, but none of them withstood multiple testing correction (see Table S7). In a sensitivity approach using only strong instruments, i.e., SNPs explaining at least 0.1% of the variance of the considered obesity-related trait, we found no significant causal relationships.

#### 2.3.3. Causal Effect of Steroid Hormones on CAD and Mediation via Obesity Traits

Finally, we estimated the total causal effects of the steroid hormones on CAD, using the same instruments as described above and the summary statistics for CAD taken from [1]. To assess the mediation effect of obesity-related traits, we also estimated the indirect effect as the product of the effect sizes of steroid hormone on the obesity-related trait and of the latter on CAD (taken from [20]). The direct effect can then be calculated as the difference between the total and indirect effects (see Methods).

**Figure 5:**
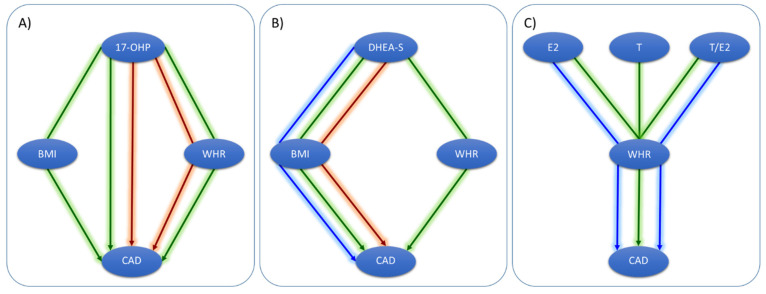
Detected causal networks of direct and indirect effects. Arrows indicate the analysis setting: green = combined; blue = males; red = females. (**A**) For 17-OHP, we detected direct and indirect effects on CAD, mediated by both BMI and WHR. (**B**) For DHEAS, we detected only indirect effects on CAD, mediated by both BMI and WHR. (**C**) For E2, T, and T/E2, we found indirect effects on CAD via WHR.

There were two significant total causal links: a negative effect of 17-OHP on CAD in both the combined setting and in females (see Table 4, columns “τ” and “p(τ)” and Figure 5). The effect was sex-unspecific (p_IA_ = 0.750). In the sensitivity analysis, the effect in females was still nominally significant, but did not survive multiple testing correction (see Table S9).

Mediation tests were restricted to the 12 causally connected pairs of steroid hormones and obesity-related traits. All related hormones had a significant indirect effect on CAD (see Table 4 columns “indir” and “p(indir)”), but only for 17-OHP, we observed significant direct effects (see Table 4 columns “dir” and “p(dir)”). Thus, all other causal relationships of hormones on CAD were mediated by obesity-related traits. As the causal effects of BMI and WHR on CAD are both positive, the directions of the indirect effect were inherited from the causal relationships of the steroid hormones on the respective obesity-related traits, e.g., a positive effect of DHEA-S and T/E2 on CAD, but negative effects of E2 and T on CAD.

Since 17-OHP was the only hormone with both, direct and indirect effects on CAD, we aimed at replicating our causal estimates considering the identified associations with HLA subtypes. This analysis confirmed that 17-OHP causally affects CAD in a sex-unspecific way (interaction *p*-values: *p* = 0.291 for the direct effect, *p* = 0.271 for the total effect, *p* = 0.149 for the indirect effect via WHR). The mediation via WHR could also be replicated. All results are summarized in Table S10.

## 3. Discussion

In the present study, we analyzed causal relationships of steroid hormones, obesity-related traits, and CAD. This was performed in a sex-stratified manner in order to contribute to the explanation of the sexual dimorphisms of these traits.

To obtain strong and valid instruments for MR analyses, we first performed sex-stratified GWAMAs of four steroid hormones: progesterone (P4), hydroxyprogesterone (17-OHP), androstenedione (A4), and aldosterone. This is an extension of our previous work [22], in which only data of one study was available for these hormones. As a novel trait of interest, we analyzed the testosterone to estradiol (T/E2) ratio. This parameter of the disturbance of the normal physiological balance of these two hormones is discussed in relation to cardiovascular disease risk [41,42]. While we successfully replicated 7 known loci, we also discovered 11 novel loci associated with these traits, of which 9 showed sex-specific effects after stringent FDR correction.

Three of these novel loci are directly linked to steroid hormone biosynthesis, namely *HSD3B1/B2* (associated with 17-OHP in males), *HSD17B7* (associated with aldosterone in females), and *CYP19A1* (associated with T/E2 in males but without differences in effect size compared to females). The *HSD3B1/B2* gene codes for 3β-hydroxysteroid dehydrogenases (two isomerases, B1 and B2) are required for the production of all biologically active steroid hormones [43]. The enzyme 17β-hydroxysteroid dehydrogenase type B7 (*HSD17B7*) is responsible for the transformation of estrone to estradiol [44], which might explain the observed female-specific effect. The link to aldosterone remains unclear so far. The hit at the *CYP19A1* gene has been previously reported for E2 [45] and T [35], but not for the ratio yet. The gene codes for the aromatase catalyze the metabolic step from T to E2.

We were able to replicate the associations at 6p21.32, 6p21.33, and 6p22.1 for 17-OHP. In our previous work [22], we did not use any fine-mapping techniques to characterize this MHC locus in more detail. Here, we used estimated HLA subtypes as an explanatory variable in a regression model for the first time and identified two of them strongly associated with 17-OHP and P4 levels, namely, HLA-C*08 and HLA-B*14, explaining the previously observed association within the MHC region. They are in LD, and HLA-B*14 might be the plausible candidate here since it has been linked to *CYP21A1* mutations [38,39] and congenital adrenal hyperplasia [46]. For our study, we excluded all participants suspected to have this autosomal recessive disorder. The observed association might be a sub-clinical sign for a disease allele carrier. Interestingly, there was a sex-specific effect on P4, but not on 17-OHP.

In our MR analyses, we used as instruments our previously published data for cortisol, DHEA-S, T and E2 [22], and our new summary statistics for 17-OHP, P4, A4, aldosterone, and T/E2. For BMI, WHR and CAD, we used publicly available summary statistics [1,13]. We detected a sex-related positive causal effect of DHEA-S on BMI, with stronger effects in females. DHEA and its sulfated ester DHEA-S are the major steroid pro-hormones in human circulation that decline with age [47]. They are transported to adipocytes [48], where DHEA is transformed to A4, which can activate the expression of androgen receptor genes [49]. Some studies have shown that DHEA reduces body fat mass in men but not women [50,51], while other trials focusing on long-term effects found no significant changes [52]. Since MR estimates the life-long causal effects of a small variation of a risk factor (due to genetics) on an outcome, its results are not necessarily comparable to clinical trials typically designed to demonstrate a short-term impact by large variations of the risk factor. As instruments for MR, we used SNPs near or within *CYP3A4* and *SULT2A1*, both catalyzing the reaction of DHEA to another metabolite, 16α-OH-DHEA and DHEA-S, respectively. In our previous work, we found sulfonation and de-sulfonation genetically regulated in females, but not males [22]. The positive effect direction we observed for DHEA-S was discordant to the above-mentioned studies regarding DHEA. Further studies regarding these sex-specific regulations of DHEA-S and their causal effect directions are required for functional validation of this mechanism.

For 17-OHP, we detected sex-unspecific causal effects on BMI, WHR, and CAD. Both direct and indirect effects on CAD, mediated via obesity-related traits were observed. The hormone was proposed as an independent predictor of WHR [53], and abdominal obesity was assumed to be associated with decreased activity of adrenal 21-hydroxylase, which is coded by *CYP21A1* in the HLA region. This is in line with our findings. In women with polycystic ovary syndrome, a positive correlation between 17-OHP and epicardial fat thickness was reported [54]. Epicardial fat thickness is related to subclinical atherosclerosis and visceral fat changes. We detected the negative causal effects of 17-OHP on CAD, both in the main analyses using SNPs and the summary statistics from van der Harst [1] and in the sensitivity analyses using HLA subtypes and only the data of our own studies. Supporting our finding, in a male rabbit model, the group on high-dose 17-OHP was found to be associated with less aortic plaques than controls, after controlling for cholesterol and triglyceride levels [55]. In summary, the causal links of 17-OHP to WHR and CAD are plausible.

Finally, we found the causal effects of E2, T, and T/E2 on WHR in both the combined setting and males. For the female subgroup, estimates could not be calculated since there were either no strong instruments for females (T, T/E2) or the statistics of the outcome could not be matched to the available instrument (E2). Hence, the sex-specificity for these links could not be tested. The effects of E2 and T alone were negative, while the hormone ratio had a positive causal effect on WHR. In a study of young women, both E2 and T were negatively correlated with WHR, but there was a significant T × E2 interaction on WHR [56]. Hence, our results are in line with previous findings, but further GWAS on T, E2, and their ratio is required to detect further strong SNPs to be used as instruments in MR.

This study was limited by the small number of instruments for the steroid hormones, which lowers the power of Mendelian randomization. However, for all hormones, instruments in or nearby genes with direct influence on the steroid hormone synthesis pathway were available, strengthening the assumption that the variants affect the analyzed outcomes via the respective hormone. A second limitation is that the summary statistics for CAD were only available for the combined setting. According to the latest CAD GWAMA [57], there are only nine sex-specific CAD loci, suggesting that the combined effects could be used for the sex-stratified analyses as well. Finally, our MR methods provide causality estimates in a statistical sense, requiring validations in experiments or randomized trials.

In conclusion, we identified 11 novel genetic loci of steroid hormone levels with pronounced sex effects. In a fine-mapping approach of the MHC region, we found two HLA subtypes significantly associated with 17-OHP and P4. Based on these loci, we discovered the sex-specific causal networks of steroid hormones, obesity-related traits, and CAD.

## 4. Materials and Methods

### 4.1. Cohort Description

Two studies of the Leipzig Research Centre for Civilization Diseases (LIFE) were analyzed: LIFE-Adult is a population-based cohort of citizens of Leipzig, Germany (*n* = 10,000) [24]. Recruitment took place from 2011 to 2016. Participants were phenotyped in detail with respect to common civilization diseases such as subclinical atherosclerosis, metabolic diseases, and cognitive function.

LIFE-Heart is a cohort of patients with suspected or confirmed coronary artery disease or myocardial infarction [23]. Patients were recruited at the Heart Center Leipzig, Germany, and all underwent coronary angiography. In the subset of patients with suspected CAD, other atherosclerotic traits were also assessed, including plaques of carotid vessels and ankle-brachial-index.

Both LIFE studies meet the ethical standards of the Declaration of Helsinki. They are approved by the Ethics Committee of the Medical Faculty of the University of Leipzig, Germany (Adult: Reg. No. 263-2009-14122009, Heart: Reg. No. 276-2005). Written informed consent, including agreement to genetic analyses was obtained from all participants.

### 4.2. Measurement of Steroid Hormones, Obesity Traits, and CAD

In LIFE-Adult, levels of the four steroid hormones—progesterone (P4), hydroxyprogesterone (17-OHP), androstenedione (A4), and aldosterone—were measured by liquid chromatography–tandem mass spectrometry (LC—MSMS) [58], while testosterone (T) and estradiol (E2) were measured by an electrochemiluminescence immunoassay (ECLIA; Roche Cobas). In LIFE-Heart, all six steroid hormones were measured by LC—MSMS.

Samples were excluded from hormone analyses if the participant was on steroid medication (ATC codes starting with “G03” or “H02AB”) or if quality control of the steroid profile suggested a mix-up of samples, or underlying diseases, e.g., hyperandrogenism, hypogonadism, adrenal insufficiency, congenital adrenal hyperplasia, or polycystic ovary syndrome.

In both studies, participants were measured for height, weight, and waist and hip girths. Based on these characteristics, BMI and WHR were calculated as obesity-related traits. All LIFE-Heart patients received diagnostic coronary angiography, and CAD was defined as at least one stenosis of ≥50% of any major coronary vessel. Both, anthropometric and CAD data were used in MR sensitivity analyses using HLA subtypes as instruments.

### 4.3. Genotyping, Imputation, and HLA Subtype Estimation

Both LIFE studies were genotyped using the Affymetrix Axiom SNP-array technology [59] (LIFE-Adult: CEU1 array, LIFE-Heart: CEU1 or CADLIFE array (customized CEU1 array containing additional SNPs from CAD loci)). Genotype calling was performed for each study with Affymetrix Power Tools (v1.20.6 for LIFE-Adult CEU1; v1.17.0 for LIFE-Heart CADLIFE; v1.16.1 for LIFE-Heart CEU1), following best practice steps for quality control. These steps comprised sample filters for signal contrast and sample-wise call rate, and SNP filters regarding platform specific cluster criteria. The datasets of LIFE-Heart typed with different array platforms were merged after calling (intersection of SNPs).

Samples with XY irregularities, including sex mismatches or cryptic relatedness, and genetic outliers (>6 SD of genetic principal components) were excluded. Further, variants with a call rate less than 0.97, Hardy-Weinberg equilibrium *p* < 1 × 10^−6^, and minor allele frequency (MAF) < 0.01 were removed before imputation. Imputation was performed using the 1000 Genomes Project Phase 3 European reference panel [25] with IMPUTE2 [60]. In summary, 7669 and 5700 samples were genotyped in LIFE-Adult and LIFE-Heart, respectively (7660 and 5688 samples for chromosome X).

To estimate the HLA subtypes, we selected all SNPs of the MHC region on chromosome 6 (25,392,021–33,392,022 Mb according to hg19, a long-range LD region) that could be matched to the Axiom HLA reference set [61]. The best-guess genotype was defined with the threshold of genotype probability >0.9, and SNPs with more than 3% missing genotype calls were excluded. Then, HLA subtypes were imputed using the Axiom HLA Analyses Tool [61,62]. A probability score was given for each sample and allele, and to filter for good quality, the combined probability was used (product of two probability scores per sample, threshold ≥0.7). In addition, we excluded HLA subtypes that were rare (<1% in each study). For every HLA subtype and sample, we estimated the dosage of each allele ranging from 0 to 2.

### 4.4. Statistical Analysis

#### 4.4.1. GWAMA

Single study GWAS. The four hormones (P4, 17-OHP, A4, and aldosterone) and the hormone ratio (T/E2) were log-transformed for all analyses to obtain normally distributed traits. We performed genome-wide association analysis for each study (GWAS) and phenotype in all samples (combined setting) and sex-stratified samples (male and female settings), with adjustment for age, log-transformed BMI, and sex in the combined setting. For analyses, we used the additive frequentist model with expected genotype counts as implemented in PLINK 2.0 [63,64].

File QC. All SNPs were harmonized to the same effect allele and were filtered for minor allele frequency (MAF) <1%, imputation info score <0.5, and minor allele count (MAC) ≤6. In addition, we checked for mismatching alleles or chromosomal position with respect to 1000 Genomes Phase 3 European reference [25] and excluded SNPs with a high deviation of study to reference allele frequency (absolute difference >0.2). Only SNPs in the intersection of both studies were meta-analyzed.

Meta analyses. For meta-analyses, single study results per phenotype and setting were combined using a fixed-effect model, assuming homogenous genetic effects across studies. We used I^2^ statistics to evaluate heterogeneity and filtered our results with I^2^ ≥ 0.9. Finally, we excluded SNPs with a minimum imputation info-score across studies of less than 0.8. The genome-wide and suggestive significance levels were set to α_gw_ = 5 × 10^−8^ and α_sug_ = 5 × 10^−6^, respectively.

Annotation. SNPs reaching at least suggestive significance for one of the phenotypes were annotated with nearby genes [65], eQTLs [66] in linkage disequilibrium (LD) r^2^ > 0.3, and known associated traits [67] in LD r^2^ > 0.3 using 1000 Genomes Phase 3 (European samples) [25] as the LD reference. We also used the genome-wide data to estimate the genetically regulated gene expression per tissue and tested for their association with our hormone levels (MetaXcan [68]).

#### 4.4.2. HLA Association

We used linear regression models to test for associations of the dosage of HLA subtypes with hormone levels. The same models as described in the GWAMA section were analyzed. There were 108 HLA subtypes available in both studies for meta-analyses. Regression models were run in R v.3.6.0. We also tested BMI, WHR, and CAD for association with HLA subtypes. Here, we used linear regression for analyses of BMI and WHR and logistic regression for analysis of CAD, and adjusted for age, log-BMI (in the WHR analysis), and sex (in the combined analysis). CAD was only available in LIFE-Heart, while BMI and WHR were available in both LIFE cohorts.

To identify independent subtypes, we estimated pairwise correlations between subtype allele dosages (i.e., Pearson’s correlation between HLA-B*14*02 and HLA-C*08*02). In addition, we looked up asymmetric LD between HLA genes (e.g., HLA-B and HLA-C). While traditional LD estimates the correlation between bi-allelic loci, asymmetric LD captures the asymmetry of multi-allelic loci [69]. We used haplotype frequencies from Wilson et al. [37], and the function compute.ALD() of the R package “asymLD” [69].

#### 4.4.3. Genetic Sex Interaction

We tested the 16 lead SNPs reaching genome-wide significance in any setting and the six significant HLA subtypes associated with steroid hormone levels regarding sex-specific effects. This was done by comparing the effect sizes of males and females for the best-associated phenotype (*t*-tests of β estimates) [70]. To adjust for multiple testing of several SNPs per hormone, we performed hierarchical FDR correction [71]. The first level of correction was the number of SNPs per hormone; the second level was the analyzed hormones.

#### 4.4.4. Mendelian Randomization (MR)

MR models. We investigated three possible causal links between steroid hormones, obesity-related traits, and CAD in a sex-specific manner. First, we tested for causal links between steroid hormones and obesity-related traits (BMI, WHR) in both directions. Then, we searched for causal links of steroid hormones on CAD and tested all significant links of steroid hormones and obesity-related traits for mediation effects on CAD by estimating direct and indirect effects (mediation MR). A graphical summary of this approach is given in Figure 1.

Data Source. As instruments for SH, we used SNPs associated with the analyzed hormones at biologically meaningful loci, e.g., genes coding for enzymes of the steroid hormone biosynthesis pathway. Statistics were obtained from the above-mentioned GWAMA and our previous work on cortisol, DHEAS, T, and E2 [22]. While sex-stratified summary statistics were available for BMI and WHR [13], this was not the case for CAD [1]. Thus, we used the combined effect estimates for all CAD analyses, i.e., we assumed no sex interactions of CAD associations. Since not all SNPs were available for all outcomes, we first used a liberal cut-off of 1×10^−6^ to get a comprehensive SNP list, and then selected for each exposure–outcome combination the best-associated SNP per locus for which outcome statistics are available. For 17-OHP, we repeated the analyses using the associated HLA subtypes as instruments to replicate our respective causal findings. As for these subtypes, association statistics for BMI, WHR, and CAD were not available in the literature; we estimated them in our LIFE studies.

Key Assumptions. SNPs were assumed to satisfy the three MR assumptions for instrumental variables (IVs): (1) The IVs were, genome-wide, significantly associated with the exposure of interest. This was shown by our GWAMA results. (2) The IVs were uncorrelated with confounders of the relationship of exposure and outcome. This might be a concern for sex, since the SNPs are partly sex-specific or sex-related, and the outcomes display sexual dimorphisms. Therefore, we ran all MR analyses in a sex-stratified manner using only those SNPs as IVs that were significant in the respective strata. (3) The IVs correlated with the outcome exclusively by affecting the exposure levels (no direct SNP effect on the outcome). Some loci are known to be associated with CAD or obesity (e.g., *CYP19A1*). However, it is highly plausible that this condition holds because we only considered loci of the steroid hormone biosynthesis pathway, which should have a direct effect on hormones.

MR Analyses. For most exposures (i.e., hormone levels), only one genome-wide significant locus was available. Hence, only one instrument was available and we applied the ratio method, which estimates the causal effect as the ratio of the SNP effect on the outcome by the SNP effect on the exposure [21]. The standard error was obtained by the first term of the delta method [21]. In the case of multiple independent instruments, we used the inverse variance weighted method to combine the single ratios [72]. To adjust for multiple testing, we performed hierarchical FDR correction per exposure [73]. First, FDR was calculated for each exposure separately. Second, FDR was determined over the best-causally related outcome per exposure. We then applied a significance threshold of α = 0.05 × k/n on the first level, with k/n being the ratio of significance to all exposures at the second level.

For mediation analyses, we used the total causal estimates α (SH → obesity-related trait), τ (SH → CAD), and β (obesity-related trait → CAD). While α and τ were calculated as described above, the causal effects of BMI and WHR on CAD were taken from [20] (Table 1). The OR and confidence intervals reported there were then transformed to effect sizes through dividing by 1.81 according to [74]. The indirect effect was estimated as the product of α and β. This product was compared with the direct effect τ by formal *t*-statistics of the differences:

(1) $${\hat{\beta }}_{\mathrm{indir}}\left(\mathrm{SH}\;\to \;\mathrm{CAD}\right)=\alpha \times \beta ,$$

(2) $$\mathrm{SE}\left({\hat{\beta }}_{\mathrm{indir}}\right)=\sqrt{\alpha^{2}\times \mathrm{SE}\left(\beta \right)+{\;\beta }^{2}\times \mathrm{SE}\left(\alpha \right)}$$

(3) $${\hat{\beta }}_{\mathrm{dir}}\left(\mathrm{SH}\;\to \;\mathrm{CAD}\right)=\tau -{\hat{\beta }}_{\mathrm{indir}}\left(\mathrm{SH}\;\to \;\mathrm{CAD}\right),$$

(4) $$\mathrm{SE}\left({\hat{\beta }}_{\mathrm{dir}}\right)=\sqrt{\mathrm{SE}{\left(\tau \right)}^{2}+\;\mathrm{SE}{\left({\hat{\beta }}_{\mathrm{indir}}\right)}^{2}}$$

## Figures and Tables

**Table 1:** Study characteristics of LIFE-Adult and LIFE-Heart. Binary variables other than sex are given in absolute counts and percentage. Continuous variables are reported by mean (SD) or median [range]. Abbreviations: CAD, coronary artery disease; BMI, body mass index; WHR, waist-hip-ratio; P4, progesterone; 17-OHP, hydroxyprogesterone; A4, androstenedione; Aldo, aldosterone; T/E2, ratio of testosterone and estradiol.

		LIFE-Adult			LIFE-Heart	
Parameter	Combined	Males	Females	Combined	Males	Females
Sex	1481	863 (58.3)	618 (41.7%)	2068	1357 (65.6%)	711 (34.4%)
Age, y	63.7 (7.8)	64.1 (7.8)	63.2 (7.7)	63.0 (10.9)	62.1 (11.1)	64.8 (10.3)
Current smoker	242 (16.7%)	148 (17.5%)	94 (15.6%)	401 (19.4%)	318 (23.4%)	83 (11.7%)
Type 2 diabetes	267 (23.9%)	180 (26.5%)	87 (19.9%)	644 (31.2%)	424 (31.3%)	220 (30.9%)
CAD	---	---	---	836 (41.9%)	646 (49.2%)	190 (27.8%)
BMI, kg/m^2^	27.9 (4.5)	28.0 (4.1)	27.7 (5.1)	29.7 (5.0)	29.5 (4.6)	30.1 (5.7)
WHR	0.96 (0.08)	1.01 (0.06)	0.89 (0.06)	0.97 (0.09)	1.02 (0.06)	0.89 (0.06)
P4 ^1^, nmol/L	0.26	0.32	0.21	0.34	0.35	0.31
	[0.20–0.37]	[0.24–0.41]	[0.16–0.32]	[0.27–0.42]	[0.28–0.44]	[0.25–0.39]
17-OHP, nmol/L	1.66	2.34	0.80	1.36	1.78	0.64
	[0.87–2.65]	[1.75–3.20]	[0.55–1.14]	[0.77–2.14]	[1.24–2.41]	[0.42–0.94]
A4, nmol/L	2.41	2.65	2.03	2.16	2.32	1.85
	[1.79–3.19]	[2.03–3.38]	[1.46–2.86]	[1.58–2.97]	[1.75–3.11]	[1.33–2.66]
Aldo, pmol/L	131	122	145	112	112	113
	[83–195]	[83–186]	[92–207]	[61–186]	[60–186]	[62–187]
T/E2 ^2^	86.3	182.5	15.2	144.5	230.0	51.6
	[16.3–187.3]	[131.2–250]	[4.7–31.0]	[66.9–353.3]	[141.6–891]	[25.3–78.7]

^1^ Using 553 additional female samples in LIFE-Adult. ^2^ Using ECLIA measurements for *N* = 5575 (2928 males, 2648 females) in LIFE-Adult.

**Table 2:** Summary statistics of novel loci. Per locus, we report the strongest association statistics for the best-associated variant. In parenthesis, we report analysis groups showing genome-wide significance (a, all; m, males; f, females). The analysis group showing the strongest significance is displayed in bold. Abbreviation: E/OA, effect/other allele; EAF, effect allele frequency (mean of LIFE-Adult and LIFE-Heart); Info, imputation info-score (minimum of LIFE-Adult and LIFE-Heart); IA-FDR, adjusted *p*-value of interaction test, marked in bold if significant. See supplementary material for justification of proposed candidate genes.

Cytoband	Lead SNP	Nearest Gene (kb)	Candidate Gene (kb)	E/OA	EAF	Info	Associated Phenotypes	β (SE)	*p* Value	I^2^	IA-FDR
1q32.2	rs138621610	*C1orf132* (0)	*CD55* (445)	A/G	0.010	0.983	P4 (a, **f**)	0.708 (0.128)	3.27 × 10^−08^	0	**4.97 × 10^−6^**
7q36.3	rs2467806	*VIPR2* (0)	*VIPR2* (0)	C/T	0.989	0.862	P4 (**a**, m, f)	−0.373 (0.068)	3.85 × 10^−08^	0.892	**1.87 × 10^−2^**
16p13.3	rs144711998	*RBFOX1* (0)	*RBFOX1* (0)	T/C	0.025	0.879	P4 (a, **f**)	0.525 (0.095)	3.30 × 10^−08^	0	**3.13 × 10^−6^**
1p12	rs947130	*HSD3BP1* (0.31)	*HSD3B1* (33)	C/T	0.731	0.922	17-OHP (a, **m**)	0.112 (0.017)	1.03 × 10^−10^	0	**5.14 × 10^−4^**
2p15	rs17014577	*FAM161A* (0)	*REL* (913)	C/T	0.143	0.991	17-OHP (a, **m**)	0.113 (0.021)	4.49 × 10^−08^	0	7.81 × 10^−2^
18q22.3	rs1430542	*RN7SL401P* (64)	*CYB5A* (442)	C/T	0.724	0.924	17-OHP (a, **f**)	−0.166 (0.028)	6.21 × 10^−09^	0	**8.21 × 10^−7^**
10q26.3	rs11311009	*MGMT* (0)	*MGMT* (0)	C/CT	0.419	0.980	A4 (a, **m**)	0.064 (0.012)	3.78 × 10^−08^	0.367	**7.06 × 10^−4^**
1q23.3	chr1:162778017	*HSD17B7* (0)	*HSD17B7* (0)	A/C	0.015	0.969	Aldo (a, **f**)	0.565 (0.097)	5.70 × 10^−9^	0.415	**9.62 × 10^−6^**
14q31.2	rs117866409	Gene desert	*CNST* (trans-eQTL)	C/G	0.040	0.919	Aldo (a, **m**)	0.275 (0.050)	3.42 × 10^−08^	0.716	**2.81 × 10^−2^**
6q26	rs73013706	*PACRG* (0)	*PARK2* (20)	G/A	0.020	0.925	T/E2 (**a**, m, f)	−0.374 (0.068)	4.42 × 10^−08^	0	7.07 × 10^−1^
15q21.2	rs727479	*CYP19A1* (0)	*CYP19A1* (0)	A/C	0.659	0.999	T/E2 (a, **m**)	−0.141 (0.015)	4.49 × 10^−21^	0.814	5.44 × 10^−2^

**Table 3:** Results from meta-analyses of the HLA subtypes on 17-OHP and P4. Significant associations are marked in bold. Allele dose associations were calculated in each study and then combined (fixed-effect meta-model). Further statistics are given in Table S6.

HLA Subtype	Phenotype	All		Males		Females		IA FDR
		Β	*p* Value	β	*p* Value	β	*p* Value	
B*14*02	17-OHP	0.543	**6.18 × 10^−20^**	0.540	**8.96 × 10^−15^**	0.543	**3.57 × 10^−7^**	9.85 × 10^−1^
	P4	0.090	1.54 × 10^−01^	0.235	**1.83 × 10^−05^**	−0.046	6.78 × 10^−1^	**2.54 × 10^−2^**
C*08*02	17-OHP	0.480	**1.12 × 10^−20^**	0.509	**2.29 × 10^−18^**	0.416	**1.63 × 10^−5^**	7.22 × 10^−1^
	P4	0.094	7.86 × 10^−02^	0.201	**1.17 × 10^−05^**	−0.009	9.24 × 10^−1^	4.88 × 10^−2^
DQA1*01*01	17-OHP	0.092	**5.09 × 10^−06^**	0.095	**3.33 × 10^−05^**	0.090	**1.86 × 10^−2^**	9.85 × 10^−1^
DQB1*05*01	17-OHP	0.095	**1.37 × 10^−05^**	0.100	**5.30 × 10^−05^**	0.086	**3.66 × 10^−2^**	9.85 × 10^−1^

**Table 4:** Results of Mendelian randomization and mediation analyses of steroid hormones on CAD via obesity-related traits. First, the causal effects of the steroid hormones on the obesity-related mediators are provided (“α”). Then, the causal effects of the hormones on CAD are provided (“τ”). The indirect effect (“indir”) is the product of α and the causal effect of the obesity-related mediator on CAD taken from Zhang et al. [20]. Finally, the direct effect of the steroid hormone on CAD is calculated as the difference between τ and the indirect effect (“dir”). Significant causal effects are displayed in bold.

Exposure	Mediator	Set	α	*p* (α)	τ	*p* (τ)	indir	*p* (indir)	dir	*p* (dir)
17-OHP	BMI	a	0.059	**1.24 × 10^−05^**	−0.108	**1.06 × 10^−02^**	0.010	**5.97 × 10^−03^**	−0.118	**5.30 × 10^−03^**
17-OHP	WHR	a	−0.063	**8.98 × 10^−06^**	−0.108	**1.06 × 10^−02^**	−0.013	**1.24 × 10^−02^**	−0.095	**2.60 × 10^−02^**
17-OHP	WHR	f	0.085	**1.16 × 10^−07^**	−0.095	**7.79 × 10^−03^**	0.018	**8.58 × 10^−03^**	−0.113	**1.93 × 10^−03^**
DHEAS	BMI	a	0.081	**7.97 × 10^−08^**	0.041	3.72 × 10^−^^01^	0.014	**3.14 × 10^−03^**	0.027	5.61 × 10^−^^01^
DHEAS	BMI	m	0.106	**7.61 × 10^−07^**	0.066	1.43 × 10^−^^01^	0.018	**4.01 × 10^−03^**	0.047	2.96 × 10^−^^01^
DHEAS	BMI	f	0.051	**2.36 × 10^−03^**	0.029	4.60 × 10^−^^01^	0.009	**2.11 × 10^−02^**	0.020	6.07 × 10^−^^01^
DHEAS	WHR	a	0.041	**8.75 × 10^−03^**	0.041	3.72 × 10^−^^01^	0.009	**4.75 × 10^−02^**	0.032	4.83 × 10^−^^01^
E2	WHR	a	−0.150	**1.13 × 10^−11^**	0.021	7.60 × 10^−^^01^	−0.031	**5.70 × 10^−03^**	0.052	4.53 × 10^−^^01^
E2	WHR	m	−0.213	**2.56 × 10^−12^**	0.020	7.60 × 10^−^^01^	−0.044	**5.47 × 10^−03^**	0.064	3.33 × 10^−^^01^
T	WHR	a	−0.136	**1.78 × 10^−05^**	−0.203	**2.76 × 10^−02^**	−0.028	**1.34 × 10^−02^**	−0.175	6.01 × 10^−^^02^
T/E2	WHR	a	0.112	**1.13 × 10^−11^**	−0.016	7.60 × 10^−^^01^	0.023	**5.70 × 10^−03^**	−0.039	4.53 × 10^−^^01^
T/E2	WHR	m	0.139	**2.56 × 10^−12^**	−0.013	7.60 × 10^−^^01^	0.029	**5.47 × 10^−03^**	−0.042	3.33 × 10^−^^01^

## Data Availability

The data presented in this study are openly available in [Zenodo] at [doi.org/10.5281/zenodo.5644896].

## Supplementary Materials

The following data are available online at https://www.mdpi.com/article/10.3390/metabo11110738/s1, Supplemental Tables S1–S10 and Supplemental Data of the GWAMA results.
