# Supplementary material for: Sex-Specific Causal Relations between Steroid Hormones and Obesity—A Mendelian Randomization Study
Source: Metabolites. 2021 Oct 28;11(11):738. doi: 10.3390/metabo11110738 (PMC8624973; doi:10.3390/metabo11110738)
Supplement: Supplementary file 1 [file metabolites-11-00738-s001.zip › SupplementalData.pdf]

Sex-specific causal relations between steroid hormones and obesity – a Mendelian Randomization Study

Pott et al.

Supplemental Data – Detailed findings of GWAMA

2.1.1. Progesterone (P4)

For P4, we detected seven loci associated with genome-wide significance, of which three were novel associations. These loci were at cytobands 1q32.2, 7q36.3, and 16p13.3. At 1q32.2 and 16p13.3 there were female-specific effects (no effect in males, interaction after hierarchical FDR correction q_{IA}= 4.97x10^{-6} for 1q32.2, and q_{IA}= 3.13x10^{-6} for 16p13.3). The association at 7q36.3 was sex-related: the lowest p-value was observed in the combined setting, but the effect in females differed significantly from that in men (β_{males}= -0.226, p_{males}= 1.14x10^{-4}; $\beta_{females}$= -0.559, $p_{females}$= 1.45x10^{-5}; q_{IA}= 0.0187). Plausible candidate genes are: *CD55* at 1q32.2 (cis-eQTL), whose gene expression is up-regulated by progesterone in mice [1]; *VIPR2* at 7q36.3 (intron modifier), coding for a receptor of the pituitary adenylate cyclase-activating polypeptide which is controlling the hormone release for ovulation [2]; and *RBFOX1* at 16p13.3 (intron modifier), which is androgen-responsive [3].

We also tested the known loci for sex-interaction and replicated the female-specific effects at *KCNH1* (1q32.2), *ARNTL* (11p15.2), and *HSD17B12* (11p11.2). In addition, we observed a novel interaction for *CYP17A1* (10q24.32), which was male-specific. Hence, all seven observed P4 associations had significant sex-interactions: five female-specific, one male-specific, and one sex-related with stronger effect in females.

2.1.2. 17-hydroxy-progesterone (17-OHP)

We found four genome-wide significant loci for 17-OHP, one known (MHC region at chromosome 6), and three novel hits at 1p12, 2p15, and 18q22.3. The first novel locus at 1p12 was male-specific (β_{males}= 0.112, p_{males}= 1.03x10^{-10}; $\beta_{females}$= -0.016, $p_{females}$= 0.571; q_{IA}= 5.14x10^{-4}) and was located near *HSD3B1*, coding for the enzyme transforming 17-OH-pregnenolone to 17-OHP. In a previous work, we only detected this locus with suggestive significance [4]. The other two loci were at 2p15 and 18q22.3, with candidate genes *REL* and *CYB5A*, respectively. Rel and P4 are part of a DNA loop, which enhances gene expression [5], while Cyb5 has a redox effector role for Cyp17A1 [6], another enzyme of the steroid biosynthesis pathway catalyzing the reaction of P4 to 17-OHP. The effect at 2p15 was sex-unspecific, while the other was male-specific (q_{IA}= 7.81x10^{-2} at 2p15; q_{IA}= 8.21x10^{-7} at 18q22.3). The known hit at MHC was sex-unspecific (q_{IA}=0.722).

2.1.3. Androstenedione (A4)

There was only one novel locus for A4 at cytoband 10q26.3, which was male-specific (q_{IA}=7.06x10^{-4}). The most likely candidate gene was *MGMT* (intron modifier). Its genetically regulated gene expression was associated with A4 in all tissues (using MetaXcan and GTex data). P4 has been reported to suppress MGMT expression in WHO grade IV human glioblastoma multiforme cell lines [7]. The androgen and progesterone receptor are closely related, and P4 in high dosages can block the androgen receptor [8,9]. This might explain the observed A4 association, but the exact mechanisms behind this are still unclear. In addition, we replicated *CYP11B2* at 8q24.3 [4]. There was no sex-interaction observed for this locus (q_{IA}=0.573).

2.1.4. Aldosterone (Aldo)

For the first time, we were able to detect genome-wide significant hits for aldosterone, one female- and one male-specific locus. The female-specific locus was at 1q23.3 within *HSD17B7*, coding for another enzyme of the steroid pathway (q_{IA}=9.62x10^{-6}). The male-specific hit is within a gene dessert, but a trans-eQTL of *CNST*, suggesting a connection to connexins, which play a role in the renin-angiotensin-aldosterone system [13] (q_{IA}=0.0281).

2.1.5. Testosterone to estradiol ratio (T/E2)

We detected two loci associated with the ratio of T to E2. Several independent SNPs (pairwise LD $r^2<0.1$) were associated at 15q21.2 with candidate gene *CYP19A1*, which has been described previously for E2 [4], T [11], sex hormone-binding globulin levels [11], and WHR [14]. While this locus showed no sex-specific effect for E2 alone [4], we detected a weak male-specific effect for the T/E2 ratio. However, this interaction did not withstand multiple testing correction ($p_{IA}=0.0272$; $q_{IA}=0.054$). The second associated locus was at 6q26 near *PARK2*. It has been reported that T and E2 prevent lymphocytes with a homozygote missense mutation in PARK2 from oxidative stressors-evoked apoptosis in juvenile Parkinson patients [15]. Here, no sex-interaction was observed ($p_{IA}=0.707$).

References

1. Zhang, Y.; Jin, S. Mitigating placental injuries through up-regulating DAF in experimental APS mice: new mechanism of progesterone. *Clin. Exp. Immunol.* **2019**, *197*, 376–386, doi:10.1111/cei.13313.
2. Apostolakis, E.M.; Riherd, D.N.; O'Malley, B.W. PAC1 receptors mediate pituitary adenylate cyclase-activating polypeptide- and progesterone-facilitated receptivity in female rats. *Mol. Endocrinol.* **2005**, *19*, 2798–2811, doi:10.1210/me.2004-0387.
3. Lieberman, A.P.; Friedlich, D.L.; Harmison, G.; Howell, B.W.; Jordan, C.L.; Breedlove, S.M.; Fischbeck, K.H. Androgens regulate the mammalian homologues of invertebrate sex determination genes tra-2 and fox-1. *Biochem. Biophys. Res. Commun.* **2001**, *282*, 499–506, doi:10.1006/bbrc.2001.4617.
4. Pott, J.; Bae, Y.J.; Horn, K.; Teren, A.; Kühnapfel, A.; Kirsten, H.; Ceglarek, U.; Loeffler, M.; Thiery, J.; Kratzsch, J.; et al. Genetic Association Study of Eight Steroid Hormones and Implications for Sexual Dimorphism of Coronary Artery Disease. *J. Clin. Endocrinol. Metab.* **2019**, *104*, 5008–5023, doi:10.1210/jc.2019-00757.
5. Hewetson, A.; Chilton, B.S. Progesterone-dependent deoxyribonucleic acid looping between RUSH/SMARCA3 and Egr-1 mediates repression by c-Rel. *Mol. Endocrinol.* **2008**, *22*, 813–822, doi:10.1210/me.2007-0432.
6. Yadav, R.; Petrunak, E.M.; Estrada, D.F.; Scott, E.E. Structural insights into the function of steroidogenic cytochrome P450 17A1. *Mol. Cell. Endocrinol.* **2017**, *441*, 68–75, doi:10.1016/j.mce.2016.08.035.
7. Atif, F.; Patel, N.R.; Yousuf, S.; Stein, D.G. The Synergistic Effect of Combination Progesterone and Temozolomide on Human Glioblastoma Cells. *PLoS One* **2015**, *10*, e0131441, doi:10.1371/journal.pone.0131441.
8. Bardin, C.W.; Brown, T.; Isomaa, V.V.; Jänne, O.A. Progestins can mimic, inhibit and potentiate the actions of androgens. *Pharmacol. Ther.* **1983**, *23*, 443–459, doi:10.1016/0163-7258(83)90023-2.
9. Raudrant, D.; Rabe, T. Progestogens with antiandrogenic properties. *Drugs* **2003**, *63*, 463–492, doi:10.2165/00003495-200363050-00003.
10. Giri, A.; Hellwege, J.N.; Keaton, J.M.; Park, J.; Qiu, C.; Warren, H.R.; Torstenson, E.S.; Kovesdy, C.P.; Sun, Y.V.; Wilson, O.D.; et al. Trans-ethnic association study of blood pressure determinants in over 750,000 individuals. *Nat. Genet.* **2019**, *51*, 51–62, doi:10.1038/s41588-018-0303-9.
11. Ruth, K.S.; Day, F.R.; Tyrrell, J.; Thompson, D.J.; Wood, A.R.; Mahajan, A.; Beaumont, R.N.; Wittemans, L.; Martin, S.; Busch, A.S.; et al. Using human genetics to understand the disease impacts of testosterone in men and women. *Nat. Med.* **2020**, *26*, 252–258, doi:10.1038/s41591-020-0751-5.

12. Jeong, H.; Jin, H.-S.; Kim, S.-S.; Shin, D. Identifying Interactions between Dietary Sodium, Potassium, Sodium-Potassium Ratios, and FGF5 rs16998073 Variants and Their Associated Risk for Hypertension in Korean Adults. *Nutrients* **2020**, *12*, doi:10.3390/nu12072121.
13. Kurt, B.; Karger, C.; Wagner, C.; Kurtz, A. Control of renin secretion from kidneys with renin cell hyperplasia. *Am. J. Physiol. Renal Physiol.* **2014**, *306*, F327-32, doi:10.1152/ajprenal.00536.2013.
14. Pulit, S.L.; Stoneman, C.; Morris, A.P.; Wood, A.R.; Glastonbury, C.A.; Tyrrell, J.; Yengo, L.; Ferreira, T.; Marouli, E.; Ji, Y.; et al. Meta-analysis of genome-wide association studies for body fat distribution in 694 649 individuals of European ancestry. *Hum. Mol. Genet.* **2019**, *28*, 166–174, doi:10.1093/hmg/ddy327.
15. Del Jimenez Rio, M.; Moreno, S.; Garcia-Ospina, G.; Buritica, O.; Uribe, C.S.; Lopera, F.; Velez-Pardo, C. Autosomal recessive juvenile parkinsonism Cys212Tyr mutation in parkin renders lymphocytes susceptible to dopamine- and iron-mediated apoptosis. *Mov. Disord.* **2004**, *19*, 324–330, doi:10.1002/mds.10670.
